# Effects of bilingual language exposure on toddlers with autism spectrum disorder

**DOI:** 10.3389/fpsyg.2024.1412339

**Published:** 2024-12-20

**Authors:** Sarah Phillips, Amelia Yanchik, Heather Jung, Peter Vietze, Leah Esther Lax

**Affiliations:** ^1^Department of Psychology, Montclair State University, Montclair, NJ, United States; ^2^CARES Clinical Services, New York, NY, United States

**Keywords:** autism spectrum disorder, language development, multilingual learners, bilingual language acquisition, early childhood

## Abstract

Research analyzing the effects of bilingual language exposure on children with autism spectrum disorder (ASD) has increased in frequency. Utilizing the Bayley Scales of Infant and Toddler Development—Third Edition, the current study analyzed the effects of bilingual language exposure and age on language development, cognitive development, and social emotional development in toddlers with ASD. Older children demonstrated higher language scores than younger children. The interaction between ASD and bilingualism did not yield statistical significance for language, cognitive, or social emotional scores; however, the interaction between age and bilingualism was found to be significant. Age may have more of an influence on language development than ASD. Children with ASD can be raised in bilingual homes without affecting long-term development.

## Introduction

It is well documented that children with autism spectrum disorder (ASD) demonstrate language delays. Studies indicate that within the first 2 years of life, children with ASD often display delays in comprehending phrases, comprehension and usage of single words, and utilization of gestures as compared with non-ASD siblings and peers (Mitchell et al., 2006). Bilingual parents may fear that exposure to two different languages can cause further delays in language and social–emotional development for their children with ASD. Recent research denotes the immense apprehension parents have in regard to teaching their child with ASD more than one language, and that professionals often suggest focusing on only one language (Ijalba, 2016; Kay-Raining Bird et al., 2012; Yu, 2013). Many mothers of children with ASD reported that, regardless of their own level of comprehension in English, they were advised by professionals such as teachers, psychologists, and healthcare providers, to only speak English with their children (Ijalba, 2016). However, parents are more effective in communicating with their children when using their native language than when using English, the majority language of their current community (Ijalba, 2016). Children whose parents mostly spoke to them in English often had difficulties participating in family conversations conducted in the parents’ native language. Parents’ limited proficiency in English may disrupt the exchange of ideas, and shorten interactions with children (Yu, 2013).

The sociocultural perspective of language development understands that language is essential to social development and acquired in social contexts (Vygotsky, 1978; Bruner, 1985).

Children learn how to socialize through language, making it important that they speak the same language as their parents (Kremer-Sadlik and Cohen, 2005). The sociocultural perspective may have particular importance for children exposed to more than one language (Lantolf and Thorne, 2006; Fahim and Haghani, 2012).

Relationships with family members are promoted through communication, and native languages serve as an important way cultural traditions and values are transmitted to children in immigrant families (Kremer-Sadlik and Cohen, 2005; Lantolf and Thorne, 2006; Yu, 2013). Newborns have been shown to discriminate between maternal native language and another language (Gervain and Werker, 2008), even showing a preference for the language they were exposed to prenatally by their mothers (DeCasper and Spence, 1986). Communication encourages intimacy, and possibly even facilitates the development of children’s attachment to their parents (Ainsworth et al., 1978).

It is conceivable that professionals believe children with ASD will have difficulty learning more than one language, considering that ASD leads to general communication delays and specific deficits in joint-attention and attention to voices (Kuhl et al., 2005). Joint-attention uses cues such as referential pointing and eye-gaze, which allow children to “map” word labels to specific objects and concepts (Parish-Morris et al., 2007). Bilingual children have the challenge of mapping a particular word, from different languages to one concept, while children raised in monolingual homes only need to map one word to that concept. Since a child with would already have trouble mapping words to concepts, it may be reasonable to think that bilingual children with ASD would have amplified delays in language acquisition and performance.

Additionally, young children with ASD have a strong preference for non-speech analog signals, as opposed to responding to infant-directed speech or “motherese” (Kuhl et al., 2005). Some children with ASD and more significant support needs, also do not elicit the mismatch negativity (MMN) response that occurs in the brain when syllables change, while typically developing (TD) children and higher functioning children with ASD do (Kuhl et al., 2005). This response is typical when there is an auditory stimulus change, and demonstrates word discrimination (Kuhl et al., 2005). It can be inferred that lower functioning children with ASD are unable to, or have trouble, discriminating between words. ASD presents unique challenges to language and communication development causing reasonable hesitancy to expose such children to more than one language. However, it is also possible that children exposed to more than one language may get redundant information that could enhance their language performance. The semantic network model of memory was proposed in the Collins and Quillian (1972). According to this theory, memories are made possible by networks of nodes (concepts) that are connected by links or associations. Applying this theory to bilingual language learning and usage, it is reasonable to see how multiple words in different languages signifying specific referents would strengthen understanding of meaning and enhance language and knowledge. Thus, children exposed to more than one language during the course of language acquisition might have stronger semantic networks.

Although there may be the fear that a multilingual home environment will further delay language acquisition, much of the current literature on the subject does not support this approach (e.g., Drysdale et al., 2015; Wang et al., 2018). Kimbrough Oller et al. (1997) determined that monolingual and bilingual children reach the same language milestones at similar ages, suggesting that bilingualism does not have a negative effect on language acquisition. Bilingualism has even been shown to moderate some delays in language and executive functioning commonly exhibited by children with ASD. Gonzalez-Barrero and Nadig (2019) found that bilingualism mitigated the effects of ASD on set-shifting, demonstrating that bilingual children ages 6–9 with ASD outperformed monolingual peers with ASD on a dimensional change card sort (DCCS) task. Peristeri et al. (2020) analyzed a matched sample of monolingual and bilingual children with ASD finding that bilingual children with ASD scored higher on measures of sustained attention, and comparable to monolingual children with ASD on all other measures of executive functions.

Dai et al. (2018) compared toddlers with ASD exposed to one language since birth to children with ASD exposed to more than one language. When compared to children with a developmental disorder other than ASD, the children with ASD performed lower on verbal skill measures, but no main effect of bilingual language exposure was found (Dai et al., 2018). A recent pilot study using a small sample of elementary aged children (ages 6–9) found no significant difference in language performance between monolingual and bilingual children with ASD, nor a language difference between bilingual children with ASD and typically developing peers (Beauchamp et al., 2020).

A recent study of bilingual Spanish-English speaking children showed no difference in receptive and expressive language or social communication between the bilingual and monolingual children in a large sample of children between 14 and 36 months of age who were participating in Early Intervention programs (Hastedt et al., 2023). In this report, the authors include an extensive review of other US-based and non-US-based studies examining the effects of bilingual language exposure on a variety of language outcomes in children. None of these studies report composite language performance, such as the Language composite on the Bayley Scales of Infant Development. Most studies also do not compare children at different ages to determine if bilingual language expose affects younger children differently than older children.

The present study was conducted to explore the effects of bilingual language exposure on language development, cognitive development, and social–emotional development in toddlers being evaluated for Early Intervention Services with a specific interest in the influence of age. The cross-sectional approach result in illustrating differences in developmental outcome for younger toddlers (under 24 months of age) and older toddlers (older than 24 months of age). It was hypothesized that (1) older toddlers would perform better on the language, cognitive, and social–emotional portions of the assessment, in comparison with the younger toddlers, (2) toddlers exposed to more than one language before the age of two would have lower language performance than those exposed to only one language before the age of two, (3) children with ASD would perform worse than typically-developing children on the cognitive and language portions of the assessment, (4) Bilingualism would affect language acquisition in toddlers with ASD.

## Methods

### Participants

Participants constituted a convenience sample and included 412 toddlers (male = 56.1%) between the ages of 15 months and 35 months recruited from several agencies in New York City that evaluate children under 36 months for possible developmental delays. The vast majority of children included in the study were New York State Medicaid-eligible. Toddlers came from diverse backgrounds and were exposed to several different languages. A total 129 of the children came from bilingual homes where two language were spoken, and 293 children from monolingual homes, representing 25 different languages (Table 1). Children were categorized as monolingual if only one language was spoken at home, even if the primary language was not English.

**Table 1:** Monolingual and bilingual frequency distribution.

Monolingual		Bilingual	
Language	Frequency	Language	Frequency
English	195	English/Spanish	74
Spanish	63	English/Bengali	5
Cantonese	9	English/Arabic	4
Bengali	7	English/Cantonese	4
Mandarin	6	English/Greek	4
Korean	3	English/Creole	4
Arabic	3	English/Tagalog	3
Greek	1	English/Hindi	3
Tamil	1	English/Hebrew	3
Russian	1	English/French	3
Polish	1	Korean/English	2
Kannada	1	English/Mandarin	2
Tagalog	1	English/Italian	2
Malay	1	English/Russian	2
Total	293	English/Polish	2
		English/Portuguese	2
		English/Urdu	2
		English/Persian	1
		Bambara/French	1
		English/Soninke	1
		English/Fante	1
		English/Punjabi	1
		English/Turkish	1
		Cantonese/Taishanese	1
		English/Japanese	1
		Total	129

The sample included 143 children who had been diagnosed with ASD using the Childhood Autism Rating Scales-2. Participants were tested prior to entry into Early Intervention. All children received the Bayley Scales of Infant Development - Third edition (Bayley-III) as part of the EI evaluation.

The study was approved by the overseeing IRB and informed consent was obtained from all parents or guardian prior to enrollment in the study.

### Assessments

#### Bayley scales of infant development-third edition (Bayley-III)

The Bayley-III is used to evaluate infant and toddler cognitive, linguistic, motor, and social–emotional development by direct observation and probing with graded tasks (Bayley, 2006). These scales show notable predictive validity with the Wechsler Preschool Primary Scale of Intelligence (WPPSI-III) (Gordon, 2004). The Bayley-III includes parent rating scales with which the parent can rate the toddler’s social–emotional behavior and adaptive behavior. The social–emotional scales are based on research by Greenspan and Shanker (2004), Greenspan et al. (1998), and Greenspan et al. (2001). The adaptive behavior scales are derived from the Adaptive Behavior Assessment System-Second Edition (Harrison and Oakland, 2003) and show excellent test validity with the Vineland Adaptive Behavior Scales (Sparrow et al., 1984).

#### Childhood autism rating scales-second edition (CARS-2)

The CARS-2 is used to rate severity of ASD in children and ranges from scores of 15 to 60. A score of 30 serves as the cutoff score for a diagnosis of autism spectrum disorder (ASD) (Schopler et al., 1980). Criterion-related validity is reported at *r* = 0.80, indicating that the CARS diagnosis was in agreement with clinical judgments. The CARS-2 has also been shown to have 100% predictive accuracy when distinguishing between groups of children with ASD and children with intellectual disability, which is superior to the commonly used ABC and Diagnostic Checklist (Teal and Wiebe, 1986).

### Procedure

One parent or parent substitute was interviewed to obtain relevant background information about the child, including: bilingual/monolingual household status; circumstances of pregnancy, labor, and delivery; relevant health status information; family background; developmental milestones; and challenging behaviors. Each child was evaluated using the Bayley-III by a licensed clinical psychologist in the child’s primary language using an interpreter when necessary. All five domains of the scale were tested either by direct observation, test probe (cognition, communication, motor skills), or by parent report (social–emotional, adaptive behavior). The diagnosis of ASD was confirmed by the psychologist, who considered the score on the CARS-2, observation of the child during the evaluation, record review, and information provided by the parent.

## Results

To evaluate the effect of bilingual exposure and age, the sample was divided into children younger than 24 months (about 2 years) and children 24 months or older, while also comparing bilinguals with monolinguals, and children with ASD with non-ASD, typically developing, children.

### Bayley-III composite language scores

**Figure 1:**
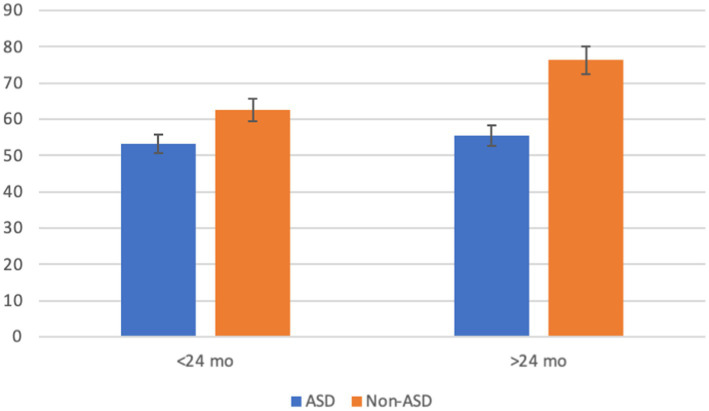
Interaction of ASD by age on Bayley-III composite language. Error bars represent 95% confidence interval.

A three-way ANOVA was conducted to analyze the main effects of age, bilingual status, and ASD and the interactions between these variables on composite language scores. The analysis did not reveal a significant interaction between age, bilingual status, and ASD (*p* = 0.183). However, the two-way interaction between age and ASD rendered a significant effect on composite language scores [*F*_(1, 334)_ = 8.1333, *p* = 0.005, *η*^2^ = 0.024] illustrated in Figure 1. Bonferroni *post hoc* tests showed a significant (*p* = 0.011) difference between young children with ASD (*m* = 53.2) and without ASD (*m* = 62.5) and a significant (*p* < 0.001) difference between older children with ASD (*m* = 55.5) and without ASD (*m* = 76.4).

**Figure 2:**
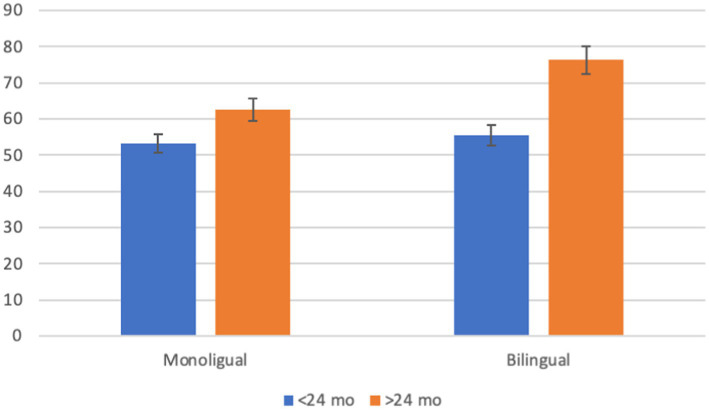
Interaction of age by bilingualism on Bayley-III composite language. Error bars represent 95% confidence interval.

There was also a significant interaction between age and bilingualism [*F*_(1, 334)_ = 3.868, *p* = 0.050, *η*^2^ = 0.011]. As shown in Figure 2, Bonferroni *post hoc* tests showed a significant difference (*p* < 0.001) between bilingual children younger than 24 months of age (*m* = 54.8) and older than 24 months of age (*m* = 66.9). The difference in age groups for monolingual children was not significant (*p* = 0.055).

### Bayley-III expressive language

The language composite scores were then broken down into expressive and receptive language scores. The factorial ANOVA analyzing the effects of age, bilingual status, and ASD on expressive language did not yield a significant three-way interaction (*p* = 0.061). A main effect for ASD [*F*_(1, 321)_ = 38.780, *p* < 0.001, *η*^2^ = 0.108] and for age [*F*_(1, 321)_ = 8.525, *p* = 0.004, *η*^2^ = 0.026] was found with small effect sizes.

**Figure 3:**
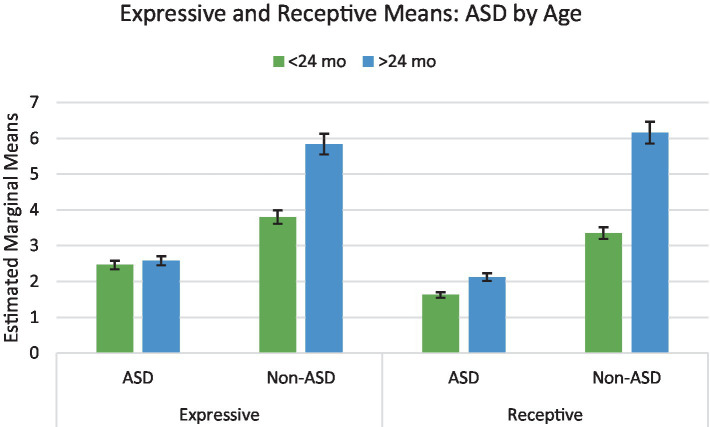
Interaction of ASD by age on Bayley-III expressive and receptive language. Error bars represent 95% confidence interval.

**Figure 4:**
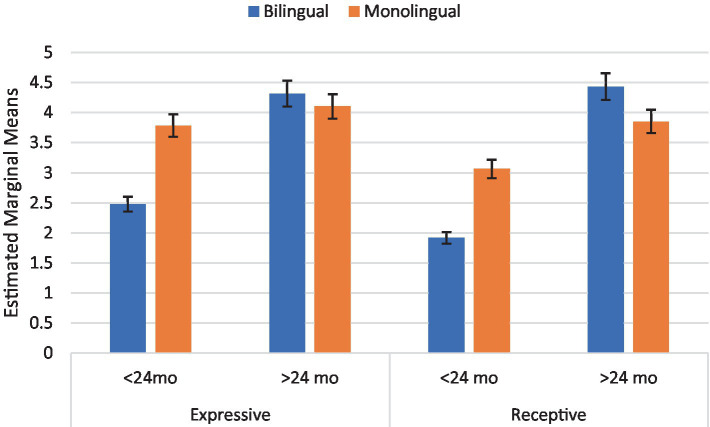
Interaction of age by bilingualism on Bayley-III expressive and receptive language. Error bars represent 95% confidence interval.

ASD and age had a significant interaction [*F*_(1, 321)_ = 6.742, p = 0.01, *η*^2^ = 0.021] demonstrating a non-significant difference in scores for the older children with ASD (*m* = 2.582) than the younger children (*m* = 2.463), yet a significantly higher score for the older typically developing children (*m* = 5.837) than their younger counterparts (*m* = 3.802) (Figure 3). There also was a significant interaction between age and bilingualism [*F*_(1, 321)_ = 4.238, *p* = 0.040. *η*^2^ = 0.013] with younger bilingual children scoring lower than monolingual peers and older bilingual children scoring slightly higher than monolingual peers. ASD and bilingualism did not have a significant interaction (*p* = 0.237) (Figure 4).

### Bayley-III receptive and expressive language

The three-way ANOVA analyzing the effects of age, bilingual status, and ASD on expressive language did not yield a significant three-way interaction (*p* = 0.061). ASD and age yielded a significant interaction [*F*_(1, 321)_ = 6.742, *p* = 0.010, *η*^2^ = 0.021]. Bonferroni *post hoc* test showed a significant (*p* = 0.039) difference between young children with ASD (*m* = 2.5) and without autism (*m* = 3.8) on expressive language scores. The interaction between bilingual status and age was also significant (*p* = 0.040). *Post hoc* tests revealed a significant difference (*p* = 0.044) between young bilingual children (*m* = 2.5) vs. monolingual children (*m* = 3.8) while older bilingual and monolingual children performed similarly (*p* = 0.550).

A similar pattern emerged for receptive language scores. The three-way ANOVA analyzing the effects of age, bilingual status, and ASD on receptive language did not yield a significant three-way interaction (*p* = 0.169), but the interaction between ASD and age [*F*_(1, 321)_ = 7.498, *p* = 0.007, *η*^2^ = 0.023] and the interaction between bilingual status and age was significant [*F*_(1, 321)_ = 4.189, *p* = 0.042, *η*^2^ = 0.013]. *Post hoc* tests demonstrated that the difference in scores between young children with ASD (*m* = 1.6) and without ASD (*m* = 3.4) was significant (*p* = 0.020) and the difference between older children with ASD (*m* = 2.1) and without ASD (*m* = 6.2) was significant (*p* < 0.001). Additionally, there was a significant difference in receptive language scores for only bilingual children between the two age groups (*p* < 0.001). Older bilingual children scored (*m* = 4.4) significantly higher than younger bilingual children (*m* = 1.9) (see Figures 3, 4).

### Bayley-III cognitive scores

**Figure 5:**
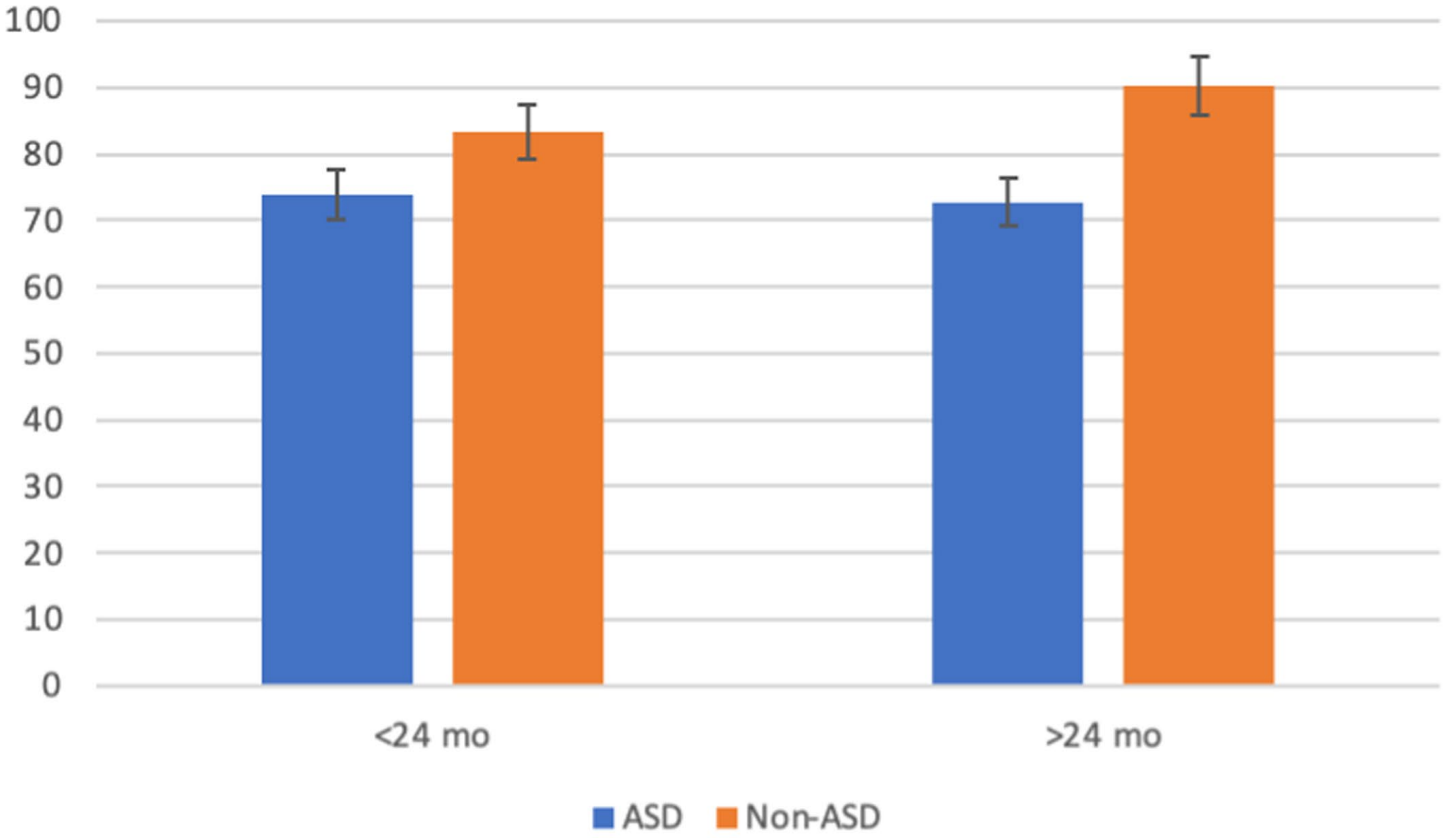
Interaction of ASD by age on Bayley-III cognitive. Error bars represent 95% confidence interval.

Similar statistical analyses were carried out for the cognitive composite scores. The three-way interaction among the independent variables was not statistically significant (*p* = 0.154). Only ASD showed a significant main effect [*F*_(1, 339)_ = 2.456, *p* < 0.001, *η*^2^ = 0.133]. As expected, children with ASD performed lower than those without ASD (*m* = 73.312, *m* = 86.815 respectively) on the cognitive measure. Bilingualism, again, did not yield a significant main effect (*p* = 0.703) or interaction with ASD (*p* = 0.435) or age (*p* = 0.205). The interaction between ASD and age was significant [*F*_(1, 339)_ = 4.603, *p* = 0.033, *η*^2^ = 0.033]. Children <24 months of age showed a significant difference in scores (*p* = 0.004) with children with ASD (*m* = 73.8) scoring lower than children without ASD (*m* = 83.3). Children older than 24 months also showed a significant difference in cognitive scores (*p* < 0.001) with children with ASD scoring lower (*m* = 72.8) than children without ASD (*m* = 90.3) (Figure 5).

### Bayley-III social–emotional scores

The three-way ANOVA analyzing the effects of age, bilingual status, and ASD on social–emotional scores did not yield a significant three-way interaction (*p* = 0.398). Only a main effect for ASD yielded a significant effect [*F*_(1, 334)_ = 58.729, *p* < 0.001, *η*^2^ = 0.150]. As would be expected, children without ASD scored (*m* = 82.2) significantly higher on the social–emotional subscale than children with ASD (*m* = 66.3).

## Discussion

The present study sought to explore the influence of language exposure among typically developing toddlers and toddlers with ASD to extend the current literature by also examining the implication age has on language development. Age and bilingual status did show significant interactions on measures of composite language, expressive language, and receptive language. There were no significant interactions with bilingual status on cognitive scores or social–emotional scores. At younger ages (< 24 months) bilingualism did affect composite language scores as well as expressive and receptive language scores among all toddlers. These deficits resolved among older toddlers (> 24 months) with bilingual toddlers scoring slightly higher than their monolingual peers. While ASD had a significant effect on both language scores (expressive and receptive), cognitive scores, and social–emotional scores bilingual status and ASD did not interact with any of the measures in the present study. Prior to 24 months children in a bilingual environment may show language delays, but older toddlers did not have the same delays resulting from bilingual language expose. Clinicians and educators may want to be causioned when suggesting that bilingual language exposure will have lasting effects on language development.

### Language outcomes

The hypothesis that bilingualism would affect language development overall, even for typically developing children, was not supported by the results. Results demonstrated that younger bilingual children scored significantly lower on the language subscale than their monolingual counterparts, performing similarly to the younger autistic children, but performed similarly to their peers when older than 24 months (about 2 years). An unintended and important finding from this analysis was demonstrating that older bilingual children scored higher on all three language measures than older monolingual children. Including the effect of age into this analysis allowed for a more nuanced look into the language development of children raised in bilingual environments.

### Cognitive outcomes

Bilingualism did not impact cognitive performance in young or older children. Some research has shown that bilingual language exposure may provide some cognitive development benefits to children as they grow older that exceed their monolingual peers. It has been demonstrated that bilingualism can positively affect executive-function development skills such as shifting between tasks, controlling attention, and expanding working memory (Bialystok and Craik, 2010). Other research suggests that speaking more than one language on a daily basis may also augment executive-functioning throughout a person’s lifetime (Bialystok and Craik, 2010; Luk et al., 2011). Furthermore, bilinguals have demonstrated advantages in certain areas of metacognitive and metalinguistic functioning (Yu, 2013). For example, children with language delays may be able to use skills developed in one language to aid in learning another language (Yu, 2013). Parents and professionals must take into consideration the ramifications of only teaching the child one language if another is primarily spoken at home.

### Social emotional outcomes

Only ASD affected social–emotional performance. Children without ASD scored higher on the social–emotional subscale than children with ASD. Bilingual status did not influence performance. Recent research has found that bilingual proficiency can show benefits on executive functioning and social–emotional outcomes (Rumper et al., 2023). Bilingualism may also be a protective factor against the negative effects of low-income neighborhoods on executive functioning and social–emotional development (Frechette et al., 2023).

### ASD and bilingualism

As expected, ASD does affect language, cognitive, and social–emotional development. No interaction between bilingualism and ASD was found suggesting that bilingual exposure will not further delay language acquisition in children with ASD or disrupt cognitive or social–emotional development. These findings contradict common suggestions from professionals to teach children with ASD only one language to avoid further language delays. According to the current study, bilingualism will not delay language development in autistic children so parents and caregivers should be encouraged to communicate with their children in their native language. Communication and the development of social skills through social interactions and verbal communication is vital for children with ASD who are already at substantial risk for social and communication deficits. Children with ASD and bilingual parents should be encouraged to communicate with their families in their parents’ native language(s). An ASD diagnosis of a child may be a profound stressor for a family. Families have reported concerns about bonding with their child because of lack of social reciprocity and communication difficulties (Norton and Drew, 1994). Adding concerns about bilingualism increasing language delays is unfounded by the current study and may only increase parental stress.

### Future directions

The results of this study are strengthened because of the use of a diverse set of bilingual language pairs (e.g., English/Spanish, Cantonese/Taishanese, English/Hebrew). The present sample consisted of children who were learning a large variety of languages and include monolingual children whose home language was not English. These methods allow the assumption that specific language pairs do not affect the results or efficacy of bilingualism on cognitive development. However, the present study did not consider the socioeconomic (SES) status of the participants and their families, though, many of the participants qualified for Medicaid. Many multilingual learners in this country are immigrants of lower SES which may limit their access to intervention services (Werker and Byers-Heinlein, 2008). Furthermore, lower SES families may not have the means of sending children to daycare or preschool, where they would have increased exposure to the majority language. SES may have had an indirect impact on the bilingual children’s outcome scores.

Age of language exposure may also have a meaningful impact on language skills. Infants 4–6 months old have demonstrated the ability to discriminate between a rhythmically different language and their native language using only visual cues but lose this ability by 8 months (Gervain and Werker, 2008). Younger infants tend to have more extensive perceptual sensitivity to stimuli such as faces and speech sounds than older infants (Pons et al., 2009). The age of secondary language exposure should be explored to understand its effect on cognitive, language, and social–emotional skills over time. It might be fruitful to study language acquisition longitudinally in toddlers with bilingual parents.

## Conclusion

Implications from this research can have an immense impact on young children, especially when, historically, parents were often instructed to teach their child with ASD only one language. While both bilingual children with ASD and typically developing bilingual children may demonstrate language delays under 24 months of age these deficits seem to resolve with age. Bilingualism was even demonstrated to provide higher language scores than monolingual peers when assessed over 24 months. Bilingualism and ASD showed no statistically significant interaction on language, cognitive, or social–emotional development. These findings should reduce the hesitancy of therapists and parents to raise children with ASD in a bilingual environment and promote parent–child communication in the family’s native language.

## Data Availability

The raw data supporting the conclusions of this article will be made available by the authors, without undue reservation.
